# Supplementary material for: The genome sequence of the Woodland Marble, Orthotaenia undulana (Denis & Schiffermüller, 1775) (Lepidoptera: Tortricidae)
Source: Wellcome Open Res. 2026 Aug 13;11:521. [Version 1] doi: 10.12688/wellcomeopenres.27389.1 (PMC13620359; doi:10.12688/wellcomeopenres.27389.1)
Supplement: Peer review history

# Reviewer report: Reviewer response for version 1

**DOI:** 10.21956/wellcomeopenres.30144.r167170

**Published:** 2026-09-28

**Authors:** Nicola J Nadeau

**Affiliations:** 1 The University of Sheffield, Sheffield, UK

This note reports the reference genome generated for the moth *Orthotaenia undulana. *Moths are ecologically important as prey for many larger species, pollinators as adults and pest herbivores as larvae. The note gives some useful background information on the ecology of this species. However, the first sentence in the introduction does not make sense and is possibly incomplete:

 "The Dusky Marble, *Orthotaenia undulana*, is a common micro-moth, although it is local in Scotland."

 This should be expanded to more clearly describe what is known about the species distribution. 

 The data note gives a detailed description of the methods, and a clear report of the data and assembly generated. The work is thorough and conducted to the recognized standards in the field. The assemblies and raw data are available in an accessible form on ENA.

 It might be useful to briefly explain what Merian elements are for non-lepidopterists. I also wonder if the Z chromosome should really be annotated as such based purely on homology, without any other supporting information (e.g. coverage in females). It would be more conservative to simply give it a number at this stage.

Are sufficient details of methods and materials provided to allow replication by others?

Yes

Is the rationale for creating the dataset(s) clearly described?

Yes

Are the datasets clearly presented in a useable and accessible format?

Yes

Are the protocols appropriate and is the work technically sound?

Yes

Reviewer Expertise:

evolutionary genetics/genomics, lepidoptera

I confirm that I have read this submission and believe that I have an appropriate level of expertise to confirm that it is of an acceptable scientific standard.

# Reviewer report: Reviewer response for version 1

**DOI:** 10.21956/wellcomeopenres.30144.r167176

**Published:** 2026-09-25

**Authors:** Eugenie C Yen

**Affiliations:** 1 Queen Mary University of London, London, UK

This manuscript presents a haplotype-resolved *de novo *genome assembly of the woodland marble ( *Orthotaenia undulana*), produced as part of the Darwin Tree of Life Project. The authors generated ~3.4 million PacBio HiFi reads and ~335 million Hi-C reads from the same male sample for phasing and scaffolding. The *de novo* assembly workflow yielded haplotype 1 curated to the chromosome level (99.5% of scaffolds assigned to 27 autosomes and the Z sex chromosome), while haplotype 2 was assembled to the scaffold level. The genome assembly stats reported in Table 2 are consistent across both haplotypes. Confirmed by the high k-mer and BUSCO completeness, the presented assembly offers a high-quality reference genome for a new Lepidopteran species to benefit biodiversity genomics research.

 The manuscript is concise yet comprehensive, featuring linked protocols, code, and clear data availability.

 I have only minor suggestions:

1. In the Background section, the common species name is referred to as the “Dusky Marble”, instead of “Woodland Marble” everywhere else. I recommend using the same common name throughout to avoid confusion.
2. (Optional) The authors might consider adding equivalent plots for haplotype 2 to Figures 5 and 6, which could be useful for those interested in using haplotype 2 in their work.

Are sufficient details of methods and materials provided to allow replication by others?

Yes

Is the rationale for creating the dataset(s) clearly described?

Yes

Are the datasets clearly presented in a useable and accessible format?

Yes

Are the protocols appropriate and is the work technically sound?

Yes

Reviewer Expertise:

Genome assembly and annotation, conservation genomics and epigenomics

I confirm that I have read this submission and believe that I have an appropriate level of expertise to confirm that it is of an acceptable scientific standard.
